# Latent tuberculosis infection in a Malaysian prison: implications for a comprehensive integrated control program in prisons

**DOI:** 10.1186/1471-2458-14-22

**Published:** 2014-01-10

**Authors:** Haider Abdulrazzaq Abed Al-Darraji, Adeeba Kamarulzaman, Frederick L Altice

**Affiliations:** 1 Centre of Excellence for Research in AIDS, Faculty of Medicine, University of Malaya, Kuala Lumpur 59990, Malaysia; 2 Department of Internal Medicine, Yale University School of Medicine, Section of Infectious Diseases, AIDS Program, New Haven, Connecticut, USA; 3 Division of Epidemiology of Microbial Diseases, Yale University School of Public Health, New Haven, Connecticut, USA

**Keywords:** Tuberculosis, HIV/AIDS, Prisons, Malaysia, Latent tuberculosis, Substance use disorders, Isoniazid preventive therapy, Integrated healthcare

## Abstract

**Background:**

Prisons continue to fuel tuberculosis (TB) epidemics particularly in settings where access to TB screening and prevention services is limited. Malaysia is a middle-income country with a relatively high incarceration rate of 138 per 100,000 population. Despite national TB incidence rate remaining unchanged over the past ten years, data about TB in prisons and its contribution to the overall national rates does not exist. This survey was conducted to address the prevalence of latent TB infection (LTBI) in Malaysia’s largest prison.

**Methods:**

From July to December 2010, all HIV-infected and a comparative group of HIV-uninfected prisoners housed separately in Kajang prison were asked to participate in the survey after explaining the study protocol. Subjects providing informed consent were interviewed using a structured questionnaire followed by the placement of tuberculin skin test (TST) with 2 TU of PPD RT-23 to subjects not being treated for active TB. TST was read after 48-72 hours and indurations of ≥ 5 mm and ≥ 10 mm were considered positive among HIV-infected and HIV-uninfected subjects, respectively. Additionally, HIV-infected inmates underwent phlebotomy for CD4 lymphocyte count assessment. A logistic regression model was explored to determine factors associated with TST positivity.

**Results:**

Overall, 286 subjects (138 HIV-infected and 148 HIV-uninfected) had complete data and TST results. The majority were men (95.1%), less than 40 years old (median age 36.0, SD 7.87), and Malaysians (93.3%). Most (82.5%) had been previously incarcerated and more than half (53.1%) reported sharing needles just prior to their incarceration. TST was positive in 88.8% (84.7% among HIV-infected and 92.5% among HIV-uninfected subjects) and was independently associated with being HIV-uninfected (AOR = 2.97, p = 0.01) and with frequent previous incarcerations (AOR = 1.22 for every one previous incarceration, p = 0.01) after adjusting for other potential confounding factors.

**Conclusions:**

The prevalence of LTBI was extraordinary high in this sample of Malaysian prisoners, regardless of their age or HIV status. This warrants further examination of the size of the problem of TB in other congregate settings and the establishment of an evidence-based TB control program in Malaysian prisons with integrated TB, HIV and substance abuse components.

## Background

Globally, prisons represent a major institutional amplifier for tuberculosis (TB), particularly in low- and middle-income countries (LMICs) [1]. The congregated environment and the concentration of TB-related risk factors among prisoners alongside the inequity in health service provision in these settings promote TB transmission and progression, particularly drug-resistant forms [2], within and beyond their walls [3]. Prisoners have a disproportionately higher prevalence of socioeconomically disadvantaged individuals, including individuals who are homeless, HIV-infected, have substance use disorders and poor access to health services compared to the non-incarcerated community [4]. Moreover, poor ventilation, overcrowding, malnutrition and psychiatric distress are prevalent in prisons and contribute and compound TB transmission among inmates in these settings [3,5]. Globally, TB among prisoners is several-fold higher than that of the general population. As such, prisons serve as reservoirs that facilitate TB transmission to the general community through released inmates and prison staff, especially where transitional care is not adequate [6,7]. Increasing incarceration rates alone has contributed to the dramatic increase in the TB incidence and prevalence of multi-drug resistant TB (MDR-TB) in the communities of European and Central Asian countries [8,9]. A systematic review of assembled reports estimated that TB exposure within prisons was attributable to 8.5% and 6.3% of all TB cases in community settings in high- and low/middle-income countries, respectively [10]. Additionally, TB contributes significantly to prison-related mortality in LMICs [4].

Malaysia, a middle-income Asia-Pacific country whose populations exceeds 29 million, has an intermediate annual TB incidence of 82 cases per 100,000 population and a TB-attributed mortality rate of 8.5 per 100,000 population [11]. In parallel, people who inject drugs (PWIDs) contribute largely to the HIV epidemic in Malaysia, where more than three-quarter of HIV-infected individuals are PWIDs [12]. While the national HIV prevalence is 0.4%, the HIV prevalence in prisons (6%), where HIV testing is mandatory, is fifteen times higher [12,13]. The high HIV prevalence among prisoners is attributed to Malaysia’s harsh criminalization laws towards drug use, including growth in prisons and compulsory drug detention centers [14,15], and placing Malaysia among the countries with the highest incarceration rate (138 per 100,000 population) worldwide [13,14].

While places like Malaysia create the ideal setting for the negative interaction among HIV, TB and incarceration, little empiric data about TB in these settings are available and provide a rationale for assessing the prevalence and correlates of latent TB infection (LTBI) among HIV-infected and –uninfected inmates in Malaysia’s largest prison.

## Methods

**Figure 1:**
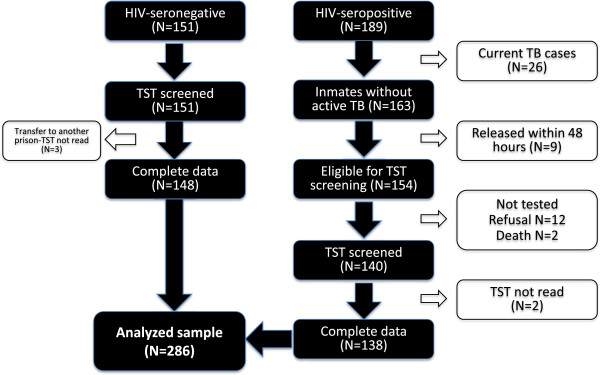
Disposition of study participants.

The cross-sectional tuberculin survey was conducted in Kajang prison, a high-security and the largest prison in Malaysia. Built to accommodate a maximum of 3500 inmates, the prison currently houses more than 4,000 inmates, operating at 119% of its actual capacity [16]. TB screening policies do not exist upon prison entry or thereafter and passive reporting of symptoms by ill inmates identifies active TB cases. Other TB control measures (e.g. TB preventive chemotherapy or infection control measures) are similarly not implemented in the prison. There are 11 separate prison units, each with different capacities, including one unit that segregates only HIV-infected prisoners. From July to December 2010, all HIV-infected inmates and a comparative group of HIV-uninfected prisoners from a similarly sized unit were voluntarily asked to participate in a tuberculin screening assessment. HIV testing is mandatory in Malaysian prisons and the 189 confirmed HIV-infected inmates in July 2010 were confined in segregated housing units in both men and women prisons. Of 189 HIV-infected prisoners, 138 had complete TST and survey data and were included in the analysis (Figure 1). A distinct housing unit for HIV-uninfected inmates was randomly selected from among 10 other units and served as a comparison group based on similar size where 151 inmates were housed. Of these, 148 had complete data. After delivering separate information sessions describing the study, interested prisoners were asked to provide informed consent. After consent, participants were interviewed using a structured questionnaire that contains sociodemographic data, incarceration history, HIV risk behaviors and any history of previous TB disease. In addition, participants were screened clinically for TB using the standardized World Health Organization (WHO) clinical scoring algorithm for prisons: cough for more than 2 weeks (2 points), sputum production (2 points), loss of weight (1 point) or appetite (1 point), and chest pain (1 point) [6]. Inmates reporting scores of 5 points or more were referred to prison health authorities for further active TB disease assessment. Due to the unavailability of TB diagnostic tools inside the prison, prisoners were referred to a nearby community hospital for further investigation, but transfer was at the discretion of prison authorities. Five HIV-infected subjects had WHO scores ≥5 had a normal chest radiograph and negative AFB microscopy and were ultimately enrolled in the study. For subjects not currently receiving active TB treatment, tuberculin skin test (TST) was placed intradermally using 2 TU RT-23 (Staten Serum Institut, Copenhagen, Denmark) and read after 48-72 hours by a single investigator (H.A.A.A). Indurations of ≥ 5 mm and ≥ 10 mm were considered positive for HIV-infected and HIV-uninfected participants, respectively [17]. All HIV-infected participants underwent phlebotomy for CD4 lymphocyte count assessment.

Data were analyzed using SPSS v19 (IBM Corporation, Somers, NY, USA). Categorical variables were presented as frequencies and continuous variables were presented by median and standard deviation (SD) or interquartile range (IQR) depending on the normality of the variable distribution. Chi-square and student t-test were utilized to compare categorical and continuous variables, respectively between the two screened groups. The primary outcome of interest was TST positivity. Covariates associated with the primary outcome on univariate logistic analysis at p < 0.20 were subsequently included in the multiple logistic regression model and controlled for potential confounders.

The study protocol was reviewed and approved by the University of Malaya Medical Centre Ethics Committee. Participation was completely voluntary and subjects who refused to participate were not in any way disadvantaged. All interviews and collected data were kept confidential.

## Results

Of the 340 recruited subjects, 286 participants (138 HIV-infected and 148 HIV-uninfected subjects) had complete data for analysis (Figure 1). The majority of participants were men (95.1%), less than 40 years old (median = 36.0, SD = 7.87 years), and ethnically Malay (93.3%). Most had completed primary education (68.8%) and were stably housed (82.1%) before being incarcerated. Nearly all were cigarette smokers (98.2%) and the majority (69.5%) drank alcohol before the current incarceration. More than half of all study participants (53.1%) reported sharing needles just prior to the current incarceration. The majority (82.5%) had been previously incarcerated for a median of 3.0 times (IQR 1-6) and a lifetime incarceration of 36 months (IQR 6-74). Nearly all (91.6%) were previously vaccinated with Bacille Calmette-Guerin (BCG) at least once, either during infancy or primary schooling, in accordance with former vaccination guidelines in Malaysia [18]. Despite being a high-risk group, the majority (92.0%) had never been previously screened for TB and 19 (6.6%) reported having received anti-TB treatment previously, but prior treatment could not be verified. Table 1 compares characteristics between HIV-infected and HIV-uninfected prisoners. Compared to those without HIV infection, HIV-infected participants were more likely to have pre-incarceration unstable housing (p = 0.005), have shared needles (p < 0.0001), have been previously incarcerated (p < 0.0001) and have been imprisoned for longer lifetime duration (p < 0.0001).

**Table 1:** Comparison of HIV-seropositive and HIV-seronegative prisoners (N = 286)

**Variables**	**Total (N = 286)**	**HIV-seropositive (N = 138)**	**HIV-seronegative (N = 148)**	**P-value**
Gender				<0.0001
Male	272	124	148	
Female	14	14	0	
Age (mean, years)	36.59	37.45	35.80	0.07
Nationality				0.02
Malaysian	281	133	148	
Foreign-borne	5	5	0	
Ethnicity				1.00
Malay	190	92	98	
Non-Malay	96	46	50	
Completed primary education				0.89
Yes	197	96	101	
No	89	42	47	
Pre-incarceration employment				0.14
No	25	16	9	
Yes	261	122	139	
Pre-incarceration stable housing				0.005
No	51	34	17	
Yes	235	104	131	
Pre-incarceration tobacco smoking				1.00
No	5	2	3	
Yes	281	136	145	
Pre-incarceration alcohol consumption				0.79
No	87	43	44	
Yes	199	95	104	
Ever shared needles before incarceration				<0.0001
No	134	12	122	
Yes	152	126	26	
BCG vaccination				0.53
No	24	10	14	
Yes	272	128	134	
Previous TB				0.001
No	267	122	145	
Yes	19	16	3	
Duration of current incarceration (mean, months)	31.87	23.23	39.76	<0.0001
Duration of the sentence served (mean, days)	182.68	160.39	203.47	0.16
Ever incarcerated				<0.0001
Yes	236	132	104	
No	50	6	44	
Number of incarcerations (mean, times)	3.9	5.4	2.5	<0.0001
Total duration of previous incarcerations (mean, months)	51.7	67.7	37.0	<0.0001

Among the HIV-infected prisoners, the median CD4 count was 384 (IQR 257-591) lymphocytes/μL; 23 (16.8%) subjects had CD4 lymphocyte count <200 cells/μL (AIDS-defining diagnosis and in need of preventive therapy against *Pneumocystis jiroveci* pneumonia) and 63 (45.6%) had CD4 ≤350 lymphocytes/μL (eligible for antiretroviral therapy; ART).

For the primary outcome, 88.8% (254 out of 286) were TST positive; 84.7% among HIV-infected and 92.5% among HIV-uninfected participants. The median size of reactive TST induration was 17 mm (SD = 6.9). After controlling for several characteristics in the bivariate analysis (see Table 2), independent correlates associated with TST positivity was being HIV-seronegative (AOR = 2.97, p = 0.01) and having been frequently incarcerated previously (AOR = 1.22 for every one previous incarceration, p = 0.01).

**Table 2:** Correlates of tuberculin skin test reactivity (N = 286)

**Variable**	**Univariate analysis**			**Multivariate analysis**		
	**OR**	**95% CI**	**p value**	**AOR**	**95% CI**	**p value**
HIV-seropositive						
Yes	Referent					
No	2.23	1.03-4.83	0.04	2.97	1.25-7.04	0.01
Age, years	1.02	0.97-1.07	0.36			
Gender						
Female	Referent					
Male	2.28	0.60-8.67	0.22			
Ethnicity						
Non-Malay	Referent					
Malay	0.75	0.33-1.69	0.49			
Completed primary education						
Yes	Referent					
No	1.17	0.52-2.65	0.70			
Pre-incarceration employment						
Yes	Referent					
No	0.92	0.25-3.25	0.89			
Pre-incarceration stable housing						
Yes	Referent					
No	0.6	0.25-1.45	0.26			
Pre-incarceration Alcohol consumption						
No	Referent					
Yes	0.89	0.39-1.99	0.76			
Current prison sentence (months)	1.01	0.99-1.03	0.12	1.01	0.99-1.03	0.11
Duration in prison currently (days)	1.00	0.99-1.01	0.79			
Ever incarcerated previously						
No	Referent					
Yes	1.68	0.71-4.00	0.24			
Number of previous incarcerations (times)	1.10	0.97-1.25	0.14	1.22	1.04-1.42	0.01
Total duration of previous incarcerations (months)	1.00	0.99-1.01	0.24			
Ever shared needles before incarceration						
No	Referent					
Yes	0.87	0.41-1.82	0.71			
BCG vaccination						
No	Referent					
Yes	1.67	0.53-5.24	0.38			

## Discussion

Globally, prisons represent major reservoirs for fuelling TB epidemics, particularly in LMICs [19]. To our knowledge, a reactive TST prevalence of 88.8% among Malaysian prisoners is the highest we have encountered in the literature to date. Moreover, we found that TST positivity was extraordinarily high, irrespective of age and HIV status.

Our findings differ markedly from other countries where economic status and national TB rates still affirm the disturbingly high prevalence of LTBI among prisoners. For example, TB surveys in other LMICs like Pakistan (48%) [20], Brazil (73%) [21] and Lebanon (45%) [22] showed high LTBI prevalence in prisons, primarily among predominantly HIV-seronegative or unknown HIV status participants, yet none of them approach LTBI prevalence found in Malaysia. Using different TST cut-offs and sampling method, TST screening among "contacts" of index TB cases with unknown HIV status in a Singaporean prison (a high-income Asia-Pacific country) showed higher prevalence of TST positivity compared to a contact screening in the community [23]. In other high-income countries, prison-based TB screening programs varied considerably, with highest LTBI prevalence in Spain (40.3% and 62%) [24,25] and Switzerland (46.9%) [26] and lowest in Italy (17.9%) [27] and the United States (17%) where HIV status was unknown [28]. The higher prevalence findings from Spain and Switzerland were partially explained by the high proportion of international migrants (foreign-born) from LMICs with high TB incidence.

While studies elsewhere have reported LTBI prevalence among prisoners in various international settings, most have not systematically assessed for independent correlates associated with LTBI. The present study expands previous findings by not only reporting the extraordinarily high LTBI prevalence among Malaysian prisoners, but provides insight into contributing factors. For example, over half of the participants in this survey were PWIDs. PWIDs represent a high-risk group for TB infection and disease progression [29] primarily due to the drug use environment itself, social and medical comorbidities and the low socioeconomic status of this population [30]. The risk is particularly high among HIV-infected PWIDs [31]. A high LTBI prevalence (ranging from 10%-59%) among PWIDs globally was recently reviewed [29] and PWIDs were concentrated among prisoners.

Prisons often lack adequate ventilation, nutrition and health services and congregate settings create an ideal environment to facilitate TB transmission to other inmates [6]. This is particularly true in settings without routine TB screening upon prison entry and thereafter. In a recent review of global screening practices in prisons, TB prevalence was found to be significantly higher in prisons without routine TB screening compared to prisons with regular screening practices (median TB prevalence of 2,227 and 343.5 cases per 100,000 population, respectively; p = 0.0059) [32]. This is in part due to lack of early detection and continued transmission to other inmates. Recently, an intensified TB case finding reported an astoundingly high prevalence (12.0%) of undiagnosed active TB disease among HIV-infected prisoners in Malaysia [33], which further supports the assumption that on-going TB transmission occurs inside Malaysian prisons and thereby contributes to the high prevalence of LTBI in this sample [5]. In the current study, the majority of subjects (82.5%) had been incarcerated at least once before and increased frequency of incarcerations was independently associated with TST positivity in this sample (AOR = 1.22 for every one previous incarceration, p = 0.01). Thus, incarceration itself contributes to increased TB acquisition.

Despite some concerns that BCG vaccination after infancy contributes to false TST reactivity [34], no association was found in this sample. The lack of association between BCG and TST positivity was confirmed among 263 health care workers (HCW) in a tertiary referral hospital in Malaysia [35]. Currently, the US Centers for Disease Control and Prevention (CDC) recommends interpretation of TST reaction regardless of the BCG vaccination history since BCG immunogenicity wanes after a few years [17].

This study corroborates earlier reports that the immunosuppressive effect of HIV infection itself impairs TST reactivity and may contribute to cutaneous anergy and potentially reduces its ability to detect LTBI [36,37]; this may be particularly true since almost half (45.6%) of the HIV-infected prisoners in this sample had a CD4 ≤350 cells/μL. Among PWIDs in the U.S., Graham *et al*. reported that having HIV infection is associated with lower TST positivity compared to HIV-uninfected patient (13.8% versus 25.5%) even when using the lower TST positivity cut-off of 5 mm, especially as CD4 counts decrease [36]. Nonetheless, anergic HIV-infected individuals remain at high risk of TB reactivation [38] and this forms the basis for the WHO recent recommendations to prescribe isoniazid preventive therapy (IPT) to all people living with HIV/AIDS (PLWHA), regardless of TST reactivity [39].

TB is recognized as the most common single cause of morbidity and mortality among HIV-infected PWIDs [40]. To properly control TB among PLWHA, WHO recommends the implementation of the "Three Is": infection control (IC); intensified case finding (ICF) and IPT, together with scaling up of ART. Given the difference in setting dynamics, the guidance recommends development of separate policies for implementing the "Three Is" in congregate settings [41]. Despite the particularly important role of IC measures in preventing TB transmission to other inmates and prison staff, most congregate settings in LMICs lack these measures [42,43]. Due to failure of current TB control strategies that mostly rely on passive case detection, ICF need to be integrated in TB control programs, particularly in correctional systems, where TB transmission is intense [44]. The lack of laboratory support and rapid point-of-care diagnostic tools complicates the feasibility of routine TB screening and implementation of ICF in prisons [32,43]. The new real-time PCR technology (GeneXpert MTB/RIF) offers a promising rapid and accurate diagnostic tool for detection of active TB disease and rifampicin resistance [45], but limited by cost, especially with the reduced diagnostic accuracy of a single specimen analysis in ICF surveys among PLWHA [33,46]. Though IPT for at least 6 months is an effective and inexpensive preventive tool, irrespective of the HIV status [47,48], the intervention needs to be thoroughly examined in correctional settings, especially with the additional barriers of limited access to diagnostic tools to exclude active TB disease, poor adherence rates post-release due to short incarceration periods, and high prevalence of co-morbidities (including HIV and hepatitis C virus infections) [7,28,49]. A recent systematic review showed a paucity in published reports on IPT use in correctional settings and the majority of reviewed studies were conducted in short-term detention centers and high-income, low TB-burden settings [7]. A shorter once-weekly isoniazid-rifapentine course for 3 months had a similar efficacy as IPT among predominantly HIV-uninfected persons in community settings [50] and warrants further investigation for treating LTBI in short-term detention centers and in patients co-infected with hepatitis C virus (HCV) and/or HIV. Moreover, the durability of this relatively short-duration treatment approach in congregate settings that facilitate TB transmission requires further exploration. A community-wide IPT implementation failed to prevent TB among gold miners in South Africa and the risk of TB started to increase shortly after the end of 9 months of IPT regimen [51], and has contributed in part to WHO’s recommendation for lifelong (or 36 months) IPT course for HIV-infected persons in high TB transmission areas [39].

In LMICs like Malaysia, the detrimental convergence of TB, HIV and substance abuse epidemics, particularly in correctional facilities, necessitates the establishment of an integrated control program targeting these co-morbidities [52]. Policy guidelines [52] and WHO recommends a "one-stop shopping" model to be instituted in similar settings where health services to HIV, TB, viral hepatitis and drug use treatment are co-located to improve access to health care, adherence to related medications and control and management of related co-morbidities [53]. The implementation of these recommendations remains minimal in countries where these measures are needed most.

Finally, finding alternatives to incarceration, particularly for PWID in countries that criminalize drug possession, may reduce TB burden in correctional settings through reductions in overcrowding of individuals at high risk for TB, a common phenomenon in LMICs prisons [54]. These measures may include drug courts, reduced bail payments and increased probation capacity [55].

**Figure 2:**
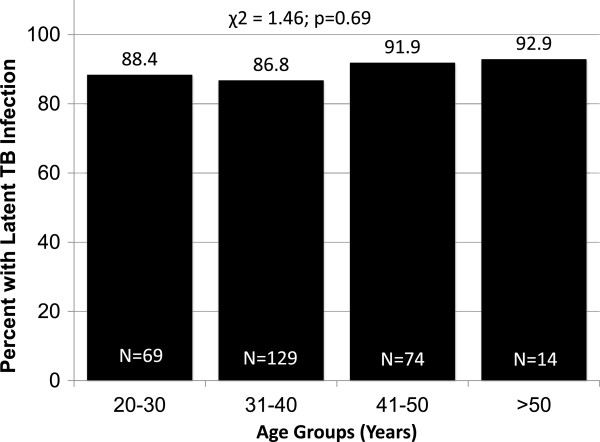
Tuberculin reactivity among different age groups.

Though the study is limited by the lack of a comparative TST survey among the Malaysian general population, the prevalence of TST positivity in this study is higher than the estimated overall LTBI prevalence (36%) in the Western Pacific region [56]. A TST survey among another high-risk group in Malaysia reported a reactive TST prevalence of 52% among predominantly young female health care workers [35]. Arguably, however, an older and predominantly male group may have higher prevalence of LTBI in an intermediate TB burden country, but the prevalence of a reactive TST was uniformly high among all screened age groups (Figure 2). Additionally, 1976 estimates of the annual risk of TB infection (ARI) in the general Malaysian population was low (0.4%) [57], which if accurate, would argue that LTBI in the community is lower than the high prevalence reported in our study.

Though the study conformed to the international guidelines of TB screening in correctional settings [6], the lack of TB diagnostic tools inside the prison and difficulties facing the referral system limited us from further assessment of active TB disease among subjects with high TST induration size, particularly among asymptomatic HIV-infected participants and should be the cornerstone of future TB screening programs.

## Conclusions

The prevalence of LTBI from the largest Malaysian prison is extraordinarily high and suggests that repeated incarcerations, particularly as a failed effort to detain PWID rather than provide rehabilitation in community settings, contribute considerably to LTBI. Findings from this study suggest the need to consider alternative approaches to incarceration in general [58] that reduce placing individuals at high risk for TB in congregate settings, but to implement an integrated TB control program that simultaneously actively screens for and treats TB, HIV and substance abuse. This should also include implementation of infection control measures, intensified TB case finding by screening upon arrival and TB preventive therapy with isoniazid or short-term preventive therapy along with evidence-based treatments for HIV and opioid dependence [40]. Further research studies are urgently needed to investigate the impact of various TB preventive measures in correctional settings.

## Abbreviations

ARI: Annual risk of infection; ART: Antiretroviral therapy; AOR: Adjusted odd ratio; BCG: Bacille Calmette-Guerin; CDC: US Centers for Disease Control and Prevention; HIV: Human immunodeficiency virus; HCV: Hepatitis C virus; HCW: Health care worker; IC: Infection control; ICF: Intensified case finding; IPT: Isoniazid preventive therapy; IQR: Interquartile range; LMIC: Low/middle-income countries; LTBI: Latent TB infection; PLWHA: People living with HIV/AIDS; PWID: People who inject drugs; TB: Tuberculosis; TST: Tuberculin skin test; SD: Standard deviation; WHO: World Health Organization.

## Competing interests

The authors declare that they have no competing interests.

## Authors’ contributions

HAAA and FLA conceptualization and designing the study; HAAA acquisition, analysis and interpretation of data; HAAA drafting the manuscript; and AK and FLA critical revision and final approval for publication. All authors read and approved the final manuscript.

## Pre-publication history

The pre-publication history for this paper can be accessed here:

http://www.biomedcentral.com/1471-2458/14/22/prepub
